# Sensitivity, specificity, positive predictive value, negative predictive value and accuracy of neuropathy diabetes score (NDS) compared with the Michigan neuropathy screening instrument (MNSI)

**DOI:** 10.1186/1758-5996-7-S1-A196

**Published:** 2015-11-11

**Authors:** Otto Henrique Nienov, Lisiane Stefani Dias, Maria Cândida Ribeiro Parisi, Helena Schmid

**Affiliations:** 1 Universidade Federal de Ciências da Saúde De Porto Alegre, Porto Alegre, Brasil

## Background

MNSI is widely used for evaluation of distal symmetric peripheral polyneuropathy (PPN) in individuals with diabetes. In the DCCT/EDIC study, the MNSI was validated for screening of signs and symptoms of PPN, presenting, for a cutoff of ≥2.5, sensitivity of 61%, specificity of 79%, positive predictive value (PPV) of 55% and negative (NPV) of 83%, when compared to neurological examination in combination with nerve conduction studies as gold standard.

## Objective

To evaluate the sensitivity, specificity, PPV, NPV and accuracy of Neuropathy Diabetes Score (NDS) (≥3.0) compared to MNSI score, used as the gold standard.

## Materials and methods

305 patients with Metabolic Syndrome, Diabetes (type 1 and type 2) were evaluated with MNSI and DNS.

## Results

**Figure 1:**
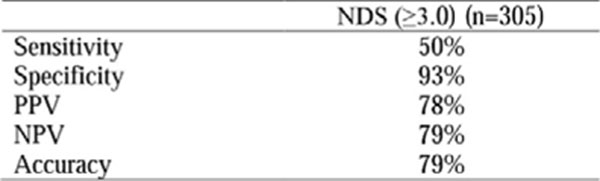
Sensitivity, specificty, PPV, NPV, and accuracy of NDS compared to MNSI.

NDS evaluates PPN signals through the thermal, painful and vibratory sensation, and the Achilles reflex. Compared to MNSI, which evaluates the PPN through the appearance of the feet, presence of ulcers, vibratory sensitivity, monofilament and Achilles reflex, NDS had a sensitivity of 50%, specificity of 93%, PPV of 78%, NPV of 79% and accuracy of 79%, according to Figure 1.

## Conclusions

When compared to MNSI as the gold standard, the NDS is a good instrument for evaluating presence of PPN, with high specificity, which reduces false positives, and good accuracy, which reflects the test precision.

